# Nitric Oxide Prevents Glioblastoma Stem Cells’ Expansion and Induces Temozolomide Sensitization

**DOI:** 10.3390/ijms241411286

**Published:** 2023-07-10

**Authors:** Luisa Salvatori, Silvia Malatesta, Barbara Illi, Maria Patrizia Somma, Cinzia Fionda, Helena Stabile, Rosaria Anna Fontanella, Carlo Gaetano

**Affiliations:** 1 Institute of Molecular Biology and Pathology, National Research Council (CNR), c/o Sapienza University of Rome, 00185 Rome, Italy; silvia.malatesta@uniroma1.it (S.M.); barbara.illi@cnr.it (B.I.); mariapatrizia.somma@cnr.it (M.P.S.); rosariaanna.fontanella@unicampania.it (R.A.F.); 2 Department of Biology and Biotechnology “Charles Darwin”, Sapienza University of Rome, 00185 Rome, Italy; 3 Laboratory Affiliated to Istituto Pasteur Italia-Fondazione Cenci Bolognetti, Sapienza University of Rome, 00161 Rome, Italy; 4 Department of Molecular Medicine, Sapienza University of Rome, 00161 Rome, Italy; cinzia.fionda@uniroma1.it (C.F.); helena.stabile@uniroma1.it (H.S.); 5 Department of Advanced Medical and Surgical Sciences, University of Campania “Luigi Vanvitelli”, 80138 Naples, Italy; 6 Laboratorio di Epigenetica, Istituti Clinici Scientifici Maugeri IRCCS, 27100 Pavia, Italy; carlo.gaetano@icsmaugeri.it

**Keywords:** glioblastoma stem cells (GSCs), glioblastoma multiforme (GBM), nitric oxide (NO), Temozolomide (TMZ), cytostasis, adjuvant treatment

## Abstract

Glioblastoma multiforme (GBM) has high mortality and recurrence rates. Malignancy resilience is ascribed to Glioblastoma Stem Cells (GSCs), which are resistant to Temozolomide (TMZ), the gold standard for GBM post-surgical treatment. However, Nitric Oxide (NO) has demonstrated anti-cancer efficacy in GBM cells, but its potential impact on GSCs remains unexplored. Accordingly, we investigated the effects of NO, both alone and in combination with TMZ, on patient-derived GSCs. Experimentally selected concentrations of diethylenetriamine/NO adduct and TMZ were used through a time course up to 21 days of treatment, to evaluate GSC proliferation and death, functional recovery, and apoptosis. Immunofluorescence and Western blot analyses revealed treatment-induced effects in cell cycle and DNA damage occurrence and repair. Our results showed that NO impairs self-renewal, disrupts cell-cycle progression, and expands the quiescent cells’ population. Consistently, NO triggered a significant but tolerated level of DNA damage, but not apoptosis. Interestingly, NO/TMZ cotreatment further inhibited cell cycle progression, augmented G0 cells, induced cell death, but also enhanced DNA damage repair activity. These findings suggest that, although NO administration does not eliminate GSCs, it stunts their proliferation, and makes cells susceptible to TMZ. The resulting cytostatic effect may potentially allow long-term control over the GSCs’ subpopulation.

## 1. Introduction

Glioblastoma multiforme (GBM) is the most prevalent primary malignant brain tumor in adults, with an exceptionally poor prognosis. With the disease essentially incurable, most patients, despite receiving a comprehensive multimodal treatment approach—encompassing surgery, radiation, and chemotherapy—inevitably succumb to tumor recurrence [1]. The relentless resurgence of the disease can be attributed to GBM’s highly invasive nature, rapid proliferation rate, and inherent resistance to standard therapies [2]. Temozolomide (TMZ), an oral alkylating agent capable of penetrating the blood–brain barrier, is currently considered the most effective therapeutic drug in our arsenal against GBM [3,4]. Nevertheless, the benefits of TMZ treatment are short-lived. The cytotoxic action of TMZ primarily stems from its ability to form O6-methylguanine in DNA, which subsequently mispairs with thymine in the ensuing DNA replication cycle. The futile cell cycles of DNA replication that follow lead to mismatch repairs, culminating in the death of cancer cells [5]. Interestingly, GBM is one of the first solid tumors where a minority population (2–3%) of stem-like, tumor-initiating cells were discovered [6]. These glioblastoma stem cells (GSCs) are believed to be the primary drivers of tumor growth. Remarkably resistant, GSCs can survive conventional oncological treatments, including TMZ, contributing to the relentless recurrence of malignancies [7,8,9]. Nevertheless, studies have demonstrated the potential effectiveness of combining chemotherapy or radiation with specific molecular agents in reducing tumor recurrence, possibly impacting the tumorigenic properties of GSCs [10,11,12]. In light of these insights, it is crucial to understand the mechanisms underpinning GSCs’ response to novel potential therapeutic agents.

Nitric oxide (NO), a signaling molecule generated by the family of nitric oxide synthases (NOS1, NOS2, and NOS3), plays a plethora of physiological and pathophysiological roles in the human body and in tumor biology [13,14]. Depending on its concentration, NO can exert opposing effects [15,16]. Indeed, under physiological conditions, low NO levels generated by NOS2 and NOS3 are cytoprotective due to antioxidant mechanisms [15,17]. Conversely, higher NO levels produced via NOS1 produce reactive nitrogen species reacting with biological targets and promoting cytotoxic/cytostatic effects [15,17]. However, several reports have shown that, at high concentrations (100–500 µM), NO induces normal cell differentiation without damaging effects. Specifically, different NO donors induce mouse embryonic stem cell differentiation in cardiomyocytes [18], mesodermic differentiation [19], osteogenic differentiation [20], as well as increase keratinocyte differentiation [21]. Importantly, NO is involved in the regulation of progenitor cells and neurogenesis in the adult rat brain [22]. It must be underlined that NO-releasing molecules have an established use in human pathologies to safely treat cardiovascular disease [23], erectile dysfunction [24], and pulmonary hypertension [25], and NO application is currently under investigation for hypertension and consequent renal failure treatment [26]. Furthermore, several clinical trials employing the use of NO supplements are currently ongoing, targeting different pathological conditions, including CNS diseases (clinicaltrials.gov website).

In the context of cancer, the influence of NO extends to cell cycle, apoptosis, mitogenic pathways, angiogenesis, invasion, and DNA integrity [27,28]. Thanks to its gaseous nature [29], NO can diffuse through cellular membranes, affecting tumor cells’ phenotype and behavior [30,31], as well as tumor microenvironment (TME) [32]. As in healthy cells, NO exhibits dual roles that are contingent on its concentration. At low levels, it can foster cancer progression, while high concentrations prove harmful to tumor survival [33,34]. Indeed, whereas NO overproduction acts as a pro-apoptotic and anti-angiogenic player, low NO concentrations are anti-apoptotic and promote angiogenesis [35,36]. In addition, low NO levels promote cell migration and invasion [37,38], while high NO concentrations reverse the epithelial-to-mesenchymal transition and the invasive phenotype of cancer cell lines [39,40]. Notably, it has been recently reported that, whereas low NO concentrations slightly increase glioma cell metabolism, higher concentrations produce a decrease. Furthermore, NO produced upon inflammatory stimuli administration has been revealed to be cytotoxic for glioma cells. Interestingly, the contrary has been observed in brain structural cells such as brain microvascular endothelial cells (BMVECs), supporting a cell-specific role for NO in the TME and suggesting a potential immunomodulatory function aimed at killing cancer cells [41]. Importantly, NO is known to cause DNA strand breaks and impede the function of DNA repair enzymes [42]. Upon incurring DNA damage, cells generally experience a temporary halt in the cell cycle and, if the damage is extensive, may ultimately undergo apoptosis in a p53-dependent process [34]. Consistently, NO influences cell cycle progression and the expression of p53 [34,43,44]. The effects of several NO donors on differentiated GBM cells have been demonstrated. For instance, S-nitroso-*N*-acetylpenicillamine (SNAP) has been shown to inhibit the growth of GBM cells both in vitro and in vivo [45]. Furthermore, treatment with SNAP or PABA/NO (O(2)-{2,4-dinitro-5-[4-(*N*-methylamino)benzoyloxy]phenyl}1-(*N*,*N*-dimethylamino)diazen-1-ium-1,2-diolate) in combination with TMZ leads to the resensitization of TMZ-resistant GBM cells [45,46]. JS-K (O^2^-(2,4-dinitrophenyl) 1-[(4-ethoxycarbonyl) piperazin-1-yl]diazen-1-ium-1,2-diolate) reduces GBM cells’ radioresistance when administered prior to radiation exposure. Various mechanisms underpin these effects, including apoptosis activation in response to DNA damage [47]. In addition, a handful of clinical studies suggest that NO donors may exert antitumor activities either as standalone treatments or in combination with standard therapies [48,49]. However, the impact of NO donors on GSCs remains largely unexplored.

Therefore, in this study, we investigated the effects of experimentally selected concentrations of NO donor diethylenetriamine/nitric oxide adduct (DETA/NO; henceforth NO) and TMZ, administered both as single treatments and in combination, on patient-derived GSCs and, when appropriate, on U87MG glioblastoma cells. Our results demonstrate that sub-lethal NO concentration can reversibly inhibit GSC proliferation and self-renewal. This finding aligns with NO-promoted DNA damage, increasing the proportion of non-proliferating cells. Intriguingly, NO also heightens the sensitivity of GSCs to TMZ treatment, primarily by amplifying cytostatic effects and inducing cell death, potentially allowing long-term control over the GSCs’ population. To the best of our knowledge, this is the first documentation of the effect of exogenous NO on GSCs, bolstering the case for NO as a potential adjunct to current GBM treatments.

## 2. Results

### 2.1. Nitric Oxide (NO) Inhibits Glioblastoma Stem Cells’ (GSCs) Expansion

**Figure 1:**
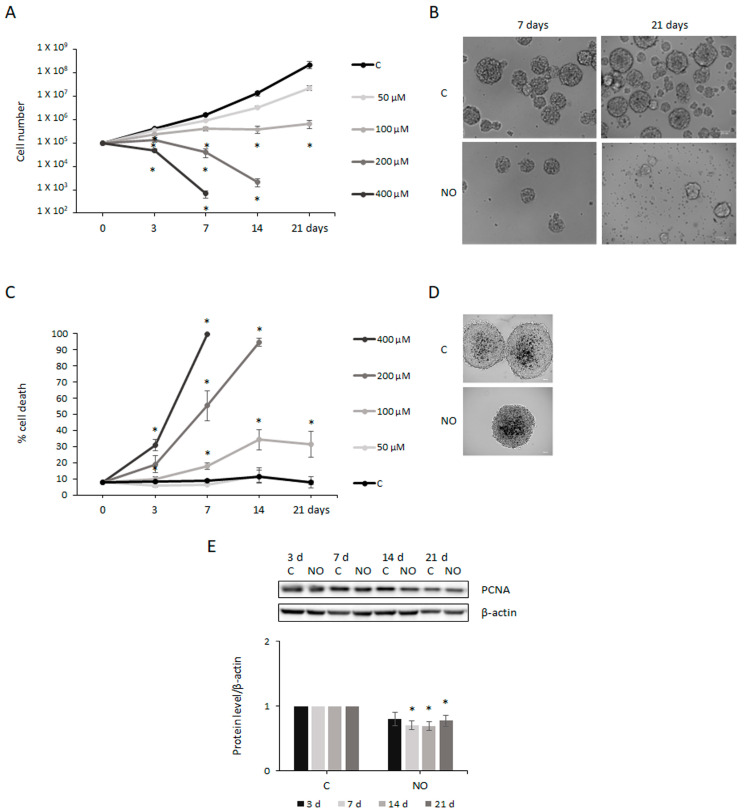
Nitric oxide (NO) inhibits Glioblastoma Stem Cells’ (GSCs) self-renewal and proliferation ability. (**A**) Proliferation of GSCs evaluated after treatment with different Diethylenetriamine/Nitric oxide adduct (DETA/NO, herein named NO) concentrations (50, 100, 200, and 400 µM) for 3, 7, 14, and 21 days. The mean ± SEM from more than 3 experiments is shown. * *p* < 0.05 vs. control cells at each time point. (**B**) Cultures of GSCs grown for 7 and 21 days in the absence (C) and presence of 100 µM NO. Photographs of neurospheres were taken under a phase contrast microscope (10×). (**C**) Cell death evaluated through Trypan blue exclusion assay following treatment with several NO concentrations and along a time course, as indicated. All the values represent the mean ± SEM from more than 3 experiments. * *p* < 0.05 vs. control cells. (**D**) Photographs show Trypan blue staining of dead cells in untreated (C) and 7 days 100 µM NO-treated neurospheres (20×). (**E**) Representative Western blot showing the expression of PCNA after 100 µM NO treatment at the indicated times. The protein level was normalized to β-actin, used as loading control, and values were shown with respect to the protein level in untreated cells at each time point (C), to which a value equal to 1 was arbitrarily assigned. The mean ± SEM of the densitometric analysis of more than 3 experiments is shown. * *p* < 0.05 vs. untreated controls.

Under appropriate culture conditions (described in Section 4), patient-derived GSCs can be maintained and expanded in vitro as floating spheres [50]. In this cell model, we tested the effects of different concentrations (50, 100, 200, and 400 µM) of NO on cell proliferation over a time course of up to 21 days. No relevant changes with respect to control cells were induced by 50 µM NO. Indeed, only a permanent slowdown in cell proliferation (Figure 1A) and no changes in cell mortality (Figure 1C) were observed. On the contrary, 200 µM and 400 µM NO caused dramatic effects on the spheres’ integrity and cell proliferation (Figure 1A), resulting in the death of all cells after 14 and 7 days of exposure, respectively (Figure 1C). These results may be ascribed to the toxic effects of high NO concentrations, as indicated by the IC50 value of 170.4 µM after 7 days of treatment. Indeed, the analysis of the effects induced by 200 µM NO exposure revealed that cell proliferation prevention was associated with a rapid and dramatic increase (about 7,5-fold with respect to untreated cells) of H2AX phosphorylation (γH2AX; Figure S1A), the most widely used marker of DNA double-strand breaks (DSBs) [51], which was consistent with the observed fast total cell mortality (Figure 1C). Moreover, a marked inhibition of PCNA, which is associated with DNA replication (−40% after 7 days of treatment; Figure S1B), and no effect on cell cycle progression, except for the reduction in the G1 phase (cell growth phase) after 3 days (Figure S1C), were observed. Interestingly, an increase in Poly (ADP-ribose) Polymerase (PARP) level, which is involved in DNA repair (Figure S1A), and a decrease in the level of the stemness marker SOX2 (−23% after 7 days of treatment; Figure S1B) were also induced. Unlike the other concentration tested, 100 µM NO induced an interesting inhibitory effect on cell proliferation. Indeed, whereas with up to 7 days of NO exposure, the number of cells progressively increased, although to a significantly lower extent than control cells, at longer times of treatment the number of cells did not rise further (Figure 1A). As shown in Figure 1B, these findings were due to the impairment of both GSCs self-renewal ability, as indicated by the reduced number of neurospheres, and cell proliferation, as spheres were smaller than those generated by untreated cells. Moreover, cells in treated neurospheres were enlarged compared with untreated spheres (Figure 1B). Consistent with cell proliferation reduction, PCNA level inhibition was observed (Figure 1E). After the stabilization of cell number, significant cell death was also induced by NO (Figure 1C,D), consistent with the increasing number of single cells (Figure 1B, right). Based on these findings, the non-toxic 100 µM NO concentration was used in all subsequent experiments to investigate long-term NO activity in GSCs. To test whether the GSCs’ response to NO exposure was cell-type-specific, we compared NO-induced effects in GSCs and GBM differentiated cells. Therefore, 100 µM NO was also tested on the U87MG cell line, a well-established glioblastoma cell model which resembles the bulk tumor. Interestingly, also in this cell system, treatment for over 7 days prevented cell population expansion (Figure S2A), but only a weak increase in cell death were observed (Figure S2B). As shown in the pictures in Figure S2C, NO treatment induced severe changes in U87MG cell morphology, as cells with long and thin cell processes or enlarged cell bodies could be observed.

### 2.2. NO Induces Reversible Effects

**Figure 2:**
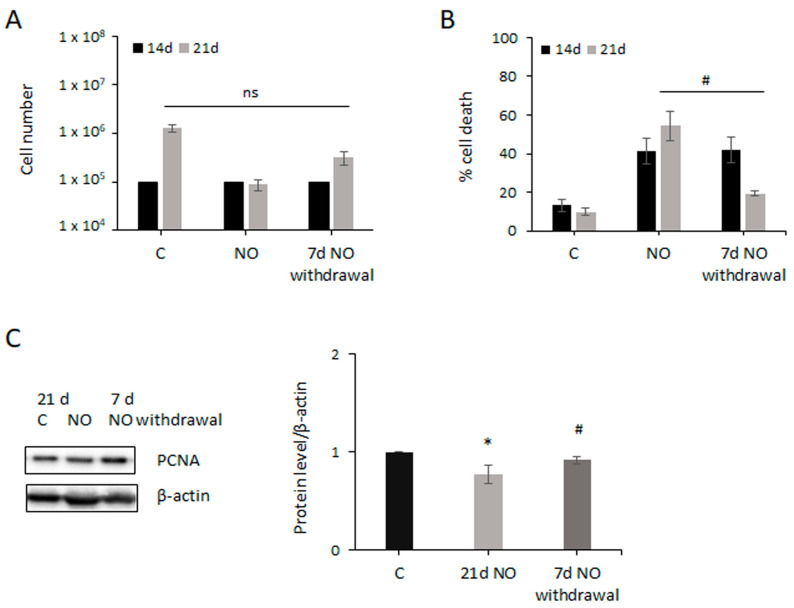

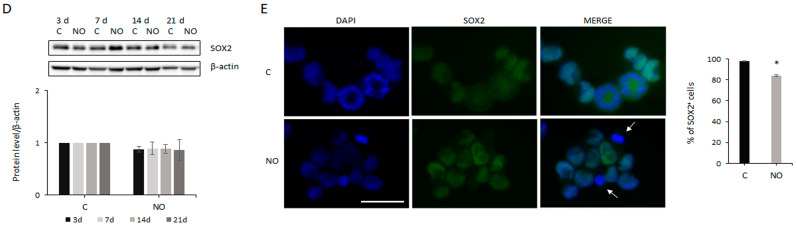
NO does not induce permanent effects and does not change GSCs’ phenotype. GSC proliferation (**A**) and death (**B**) after 14 days of NO exposure followed by 7 days of treatment withdrawal (right bars), compared to untreated cells (left bars) and cells treated for 21 days with NO (middle bars). Results represent mean ± SEM from 3 experiments. # *p* < 0.05 vs. NO-treated cells. (**C**) Representative Western blot showing PCNA expression in untreated cells (C), cells treated for 21 days with NO, and cells in which treatment was withdrawn for 7 days after 14 days of NO exposure. The graph shows the mean ± SEM of the densitometric analysis of at least 3 experiments, where PCNA was normalized to β-actin level. * *p* < 0.05 vs. untreated cells; # *p* < 0.05 vs. NO-treated cells. (**D**) Representative Western blot showing SOX2 expression after NO exposure at the indicated times. The expression level of the protein was normalized to β-actin. Line chart shows the mean ± SEM of the densitometric analysis of 3 experiments. (**E**) Immunofluorescence performed on GSCs untreated (C) and after 7 days of NO treatment. Nuclei stained with DAPI (blue), SOX2 expression (green) as well as merged images are shown. The scale bar = 50 µm is the same in every image. Arrows indicate SOX2^−^ cells. The graph shows the percentage of cells expressing SOX2 in the two experimental conditions. The values were obtained from the analysis of about 500 cells in 50 fields for each sample. All the values represent the mean ± SEM. * *p* < 0.05 vs. untreated cells.

We wondered whether long-term exposure to NO could permanently affect GSCs’ behavior. To answer this question, NO was withdrawn after 14 days of treatment, when cell expansion impairment was firmly established, and GSCs were allowed to grow for an additional 7 days in basal culture conditions. Interestingly, NO-deprived GSCs started to proliferate again similarly to untreated cells (four-fold difference between untreated and NO-deprived cells), whereas the number of NO-treated cells did not increase over time (Figure 2A). Consistently, the mortality of NO-deprived cells dramatically decreased, whereas it remained high in GSCs treated with NO for 21 days (Figure 2B). In addition, PCNA levels also increased towards control levels after NO withdrawal (Figure 2C). On the contrary, when the effect of 7 days of NO withdrawal was tested on U87MG cells previously treated with NO for 14 days, we observed a weak increase in cell proliferation (eight-fold difference between untreated and NO-deprived cells; Figure S3A) and no reduction in the percentage of cell death (Figure S3B). The observed prompt recovery of GSC proliferation led us to hypothesize that long-term NO treatment did not change the GSCs’ phenotype. In fact, Western blot analysis showed that NO administration only slightly reduced the level of the stemness marker SOX2 (Figure 2D). Consistently, immunofluorescence analysis revealed that almost all NO-treated cells maintained the expression of SOX2 but a small population of SOX2^−^ cells (about 13% of the total) was also present (Figure 2E).

### 2.3. NO Interferes with Cell Cycle Progression

**Figure 3:**
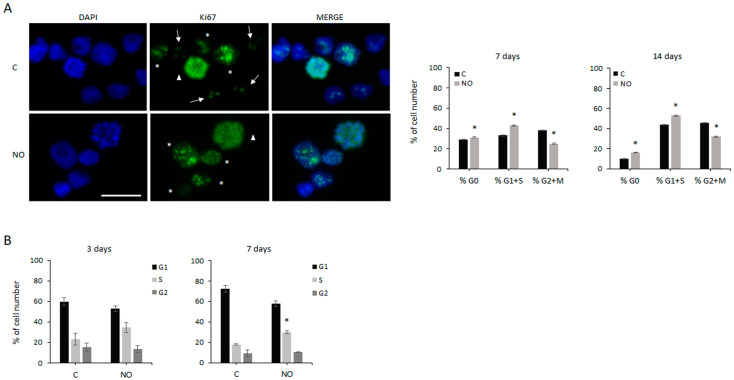
NO induces GSCs’ S phase accumulation. (**A**) Representative immunofluorescence images showing GSCs untreated (C) and after 14 days of NO exposure (NO). The same analysis was also performed after 7 days of NO administration. Nuclei stained with DAPI, Ki67 expression, and merged images are shown. Ki67 staining patterns indicate different stages of the cell cycle: asterisks indicate cells in G1/S phases, arrows indicate cells in G2 phase, and triangles indicate cells in mitosis. The scale bar = 50 µm is the same in every image. The adjacent graphs show the changes induced after 7 and 14 days of NO treatment on the percentage of cells in different phases of the cell cycle and in G0 (Ki67^−^ cells). The values were obtained from the analysis of at least 15 fields and 100–150 cells for each sample. All the values represent the mean ± SEM. * *p* < 0.05 vs. untreated control cells. (**B**) Flow cytometry analysis of the cell cycle progression after 3 and 7 days of NO exposure. Results represent the mean ± SEM from 3 experiments. * *p* < 0.05 vs. untreated control cells.

To investigate the effects induced by NO on cell proliferation, we evaluated possible changes in cell cycle progression. To this aim, we analyzed the expression of the nuclear protein Ki67, which is widely used as a proliferation marker, using immunofluorescence analysis. Indeed, quiescent cells do not express Ki67, which shows typical patterns in different cell cycle phases [52]. In particular, during the G1 and S phases, Ki67 is mostly aggregated in many small foci, whereas the G2 phase is characterized by rare and large Ki67 foci usually corresponding to nucleoli, and during mitosis Ki67 traces chromosomes. The analysis of Ki67 patterns in GSCs showed that after 7 and 14 days of NO exposure, the number of cells in the G1/S phases increased (up to +30%), whereas the number of cells in G2/M was reduced (−30%; Figure 3A) compared with untreated cells. In parallel, the number of Ki67^−^ cells increased to +60% after 14 days of NO administration. To further investigate the NO-dependent GSCs’ cell cycle regulation, cells were also analyzed with FACS. Consistent with the distribution of Ki67 foci, accumulation of GSCs in the S phase after 3 and 7 days of NO treatment was revealed (Figure 3B).

### 2.4. NO Induces DNA Damage

**Figure 4:**
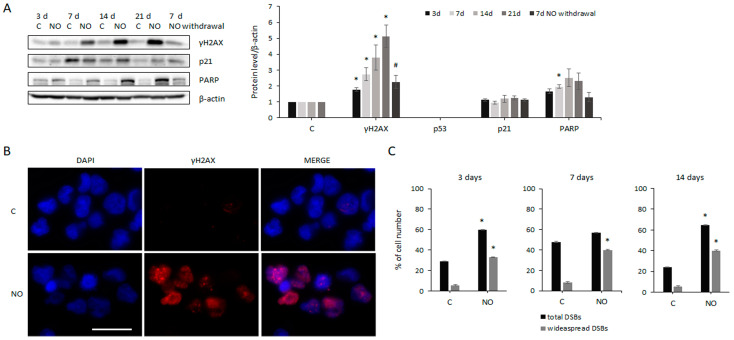
Effects of NO on GSCs’ DNA integrity. (**A**) Representative Western blots showing the expression of several markers of the DNA damage response in untreated cells (C) as well as after 3, 7, 14, and 21 days of NO administration (NO) and after 14 days of NO exposure plus 7 days of treatment withdrawal (right lane). The level of each protein was normalized to β-actin. Line chart shows the fold increase in the level of each protein after NO treatment calculated with respect to untreated cells at each time point, to which a value equal to 1 was arbitrarily assigned. Values represent the mean ± SEM of the densitometric analysis of at least 3 experiments. * *p* < 0.05 vs. control cells; # *p* < 0.05 vs. 21 days NO-treated cells. (**B**) Representative immunofluorescence staining showing GSCs untreated (top) and after 14 days of NO treatment (bottom). Nuclei, γH2AX expression, and merged images are shown. The scale bar = 50 µm is the same in every image. The same analysis was also performed on cells treated with NO for 3 and 7 days. (**C**) Graphs show the percentage of all the cells containing DSBs and those containing only widespread DSBs in NO-treated and untreated cells. The mean ± SEM of the values obtained from the analysis of at least 15 fields and 100–150 cells for each sample is shown. * *p* < 0.05 vs. control cells.

NO interference in cell cycle progression may be associated with the onset of DNA damage [43,53]. To investigate this hypothesis in our cell system, we evaluated the phosphorylation of histone H2AX. Western blot analysis showed that, whereas untreated cells had negligible γH2AX expression, histone phosphorylation progressively increased during long-term NO exposure (Figure 4A). Consistently, immunofluorescence analysis revealed that about 30% (on average) of control cells presented γH2AX staining (Figure 4B,C). In particular, most of these cells showed scarce, small, and faint γH2AX foci, which sometimes also appeared as single dots. However, a low percentage of these cells (20% of γH2AX-positive cells) had diffused and intense γH2AX staining (Figure 4B,C). Following NO treatment, the percentage of GSCs showing γH2AX staining increased about two-fold, on average (Figure 4C). Of particular interest was the observation that NO induced also a sharp enhancement of the number and the size of γH2AX foci, resulting in a marked increase (up to 70%) in the percentage of γH2AX-positive cells presenting widespread and intense γH2AX staining (Figure 4B,C). Collectively, immunofluorescence analysis revealed that NO treatment increased the percentage of GSCs with widespread and marked DNA damage from 6% (in control cells) to 40% (in treated cells). Next, we evaluated the expression of several proteins involved in DNA damage response and repair, which may be activated downstream of H2AX phosphorylation. As is already known, p53 and p21 may be sequentially involved in cell cycle arrest and apoptosis following DNA damage [43], and p21 may also participate in the DNA repair process [54]. However, Western blot analysis revealed the absence of p53 expression in GSCs (Figure 4A) and the lack of effect of NO exposure on p53 and p21 levels (Figure 4A). Differently, NO treatment induced PARP expression over time (Figure 4A). We asked whether the DNA damage induced in the GSCs was permanent or transient. Therefore, after 14 days, NO treatment was stopped, and the levels of DNA damage and repair markers were evaluated 7 days later. Interestingly, NO withdrawal dramatically reduced the level of both γH2AX and PARP (Figure 4A). Next, NO effects on DNA integrity were evaluated in U87MG cells, where a different scenario was revealed (Figure S4). Indeed, a progressive increase in H2AX phosphorylation and p53 expression was observed, as well as a p21 increase which remained stable over time. Differently, PARP level weakly increased only after the longest period of NO treatment. Seven days of treatment withdrawal after 14 days of NO administration caused a partial decrease in γH2AX and p53 levels, and a sharp rise in PARP level (Figure S4).

### 2.5. NO Does Not Induce Apoptosis

**Figure 5:**
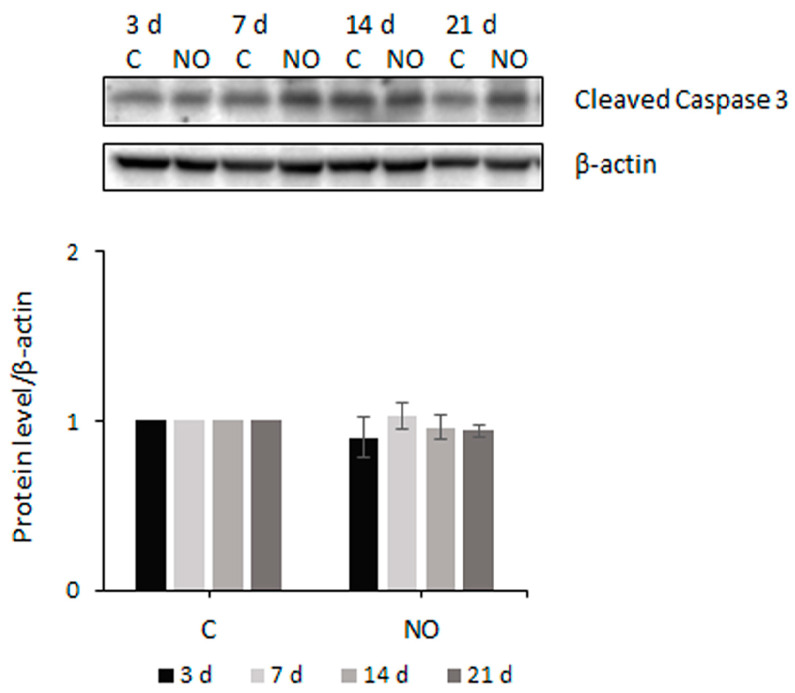
NO has no effect on caspase 3 in GSCs. Representative Western blot showing the expression of cleaved caspase 3 in GSCs untreated (C) and NO-treated for the indicated times. The level of the active caspase 3 was normalized to β-actin and represented in the graph with respect to the level measured in untreated cells at each time point, to which a value equal to 1 was arbitrarily assigned. The mean ± SEM of the densitometric analysis of 3 experiments is shown.

We asked whether apoptosis could participate in NO-induced cell proliferation impairment. No evidence of nuclear integrity loss was observed by immunofluorescence staining (Figure 2E and Figure 3A), and none of a sub-G1 population was revealed by FACS analysis (Figure 3B). Since the analysis of apoptosis-induced translocation of annexin V to the cell surface confirmed the absence of apoptotic cells following NO exposure, we evaluated the possible activation of caspase 3, a key marker of the apoptotic cascade [55]. Unexpectedly, we observed that the 17 kDa active form of the protein was constitutively expressed in untreated GSCs (Figure 5). However, its level did not increase after NO treatment. Notably, the level of cleaved caspase 3 did not increase also after 3 and 7 days of 200 µM NO treatment when massive GSCs’ mortality and DNA damage were observed (Figure 1C and Figure S1A). Similarly to the GSCs, the analysis of U87MG cells revealed a weak expression of the active form of caspase 3, which did not increase after NO treatment (same as in Figure 5).

### 2.6. Combined NO Plus Temozolomide (TMZ) Treatment Further Inhibits GSC Proliferation

**Figure 6:**
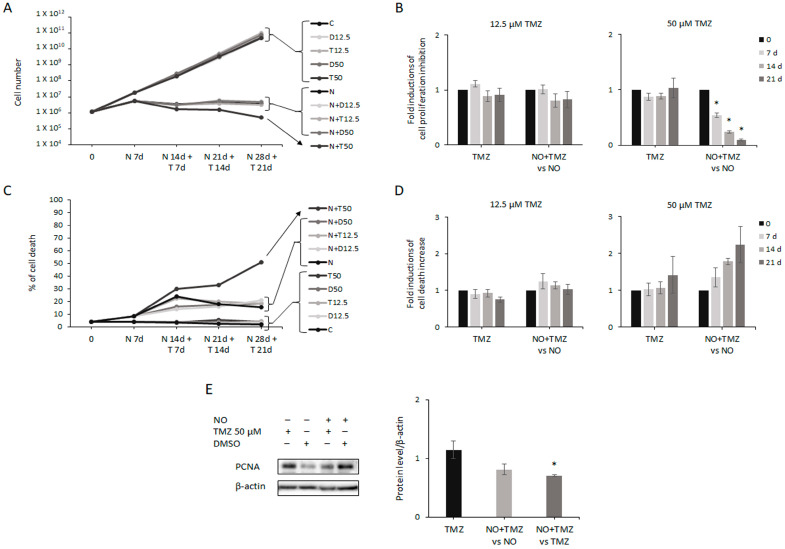
NO plus Temozolomide (TMZ) cotreatment further reduces cell proliferation. (**A**) Representative experiment showing the proliferation of GSCs evaluated after 7 days of treatment with 100 µM NO alone (N) and followed by combined treatment with NO and two concentrations (12.5 and 50 µM) of TMZ (N + T), or DMSO (N + D) at the same dilutions, for 7, 14 and 21 days. TMZ (T) and DMSO (D) were also tested on untreated cells (C), as a control. (**B**) The graphs show the effect of TMZ alone, normalized to DMSO, and the effect of the combined treatment with respect to NO administration alone on GSC proliferation. The mean ± SEM of at least two experiments is represented. * *p* < 0.05 vs. NO-treated cells. (**C**) Representative experiment showing GSCs’ death following exposure to NO (N), TMZ (T), DMSO (D), and NO plus TMZ (N + T) or NO plus DMSO (N + D), as indicated, and following the same experimental plan as described in panel (**A**). (**D**) The graphs show GSCs’ death upon TMZ administration alone and upon combined treatment analyzed with respect to NO treatment alone. Each bar represents the effect of TMZ normalized to DMSO. (**E**) Representative Western blot showing the effect of 21 days of 50 µM TMZ and DMSO administration, alone and in the presence of NO, on PCNA expression. The graph shows the densitometric analysis of the bands where TMZ’s effect was evaluated with respect to DMSO (left bar), cotreatment-induced changes were analyzed with respect to NO single administration (middle bar) as well as further normalized to TMZ (right bar). The mean ± SEM of two experiments is shown. * *p* < 0.05 vs. TMZ-treated cells.

Based on previous reports showing that NO can sensitize cells to other treatments [56,57,58], we wondered whether NO-treated GSCs, which accumulated noticeable DNA damage able to arrest cell proliferation, could be susceptible to other stimuli. Since TMZ is the first-in-line chemotherapeutic agent currently used to treat GBM, although it does not hit the GSCs subpopulation [7,8,9,59], we decided to investigate the possible effects of NO plus TMZ cotreatment in GSCs. To be consistent with clinical results, we needed to identify TMZ doses ineffective in GSCs and effective in U87MG cells. To such an aim, GSCs were exposed to several concentrations of TMZ (12.5–25–50–100–200–400–600 µM) for 3, 7, and 14 days. We found that 12.5 µM, 25 µM, and 50 µM TMZ did not reduce GSC proliferation, and also the percentage of dead cells was similar in treated and untreated cells (Figure S5A,B). Of note, prolonged treatment did not improve the effects observed after 3 days of TMZ exposure. Differently, 100 µM, 200 µM, 400 µM, and 600 µM TMZ induced a dose- and time-dependent inhibition of proliferation paralleled by a progressive increase in cell death (Figure S5A,B). The three TMZ concentrations ineffective in GSCs (12.5–25–50 µM) were then tested in U87MG cells for 3, 7, and 10 days, to confirm the known drug efficacy on differentiated bulk tumor cells [60,61]. As expected, U87MG cells showed a strong sensitivity to all the concentrations of TMZ, and cell proliferation was dose- and time-dependently reduced (Figure S5C). Consistently, cell death was progressively enhanced (Figure S5D). According to the above-described findings, showing that NO’s effects on GSCs were steadily established after 7 days of treatment, two doses of TMZ (12.5 and 50 µM), which were ineffective when given alone, were tested for 7, 14, and 21 days in combination with NO in GSCs previously treated with NO alone for 7 days. In particular, 50 µM TMZ was chosen as it was the highest concentration among the ineffective ones, while 12.5 µM TMZ was considered a possible negative control as it was a low concentration unlikely to be effective even after combined treatment. Interestingly, NO plus 50 µM TMZ cotreatment progressively reduced the number of GSCs with respect to prolonged NO treatment alone (Figure 6A,B). Accordingly, the level of PCNA was reduced by cotreatment with respect to NO single administration (Figure 6E, middle bar). The inhibitory effect of NO plus 50 µM TMZ cotreatment on cell proliferation was paralleled by a weak but progressive increase in cell death, with respect to NO-induced effect (Figure 6C,D). On the contrary, NO plus 12.5 µM TMZ combined treatment had no further effect compared to NO administration alone on both cell proliferation (Figure 6A,B) and cell death (Figure 6C,D). In agreement with the results shown in Figure S5A,B, prolonged treatment of GSCs for up to 21 days with both TMZ concentrations (in the absence of NO) did not induce any change in cell proliferation and cell death rate (Figure 6A–D). Consistently, 50 µM TMZ exposure alone induced negligible changes in PCNA expression (Figure 6E, left bar). Nevertheless, TMZ plus NO cotreatment caused a significant decrease in protein expression compared to TMZ single administration (Figure 6E, right bar). The lack of efficacy of 12.5 µM TMZ was not shown.

### 2.7. TMZ Enhances NO-Induced Inhibition of Cell Cycle Progression and Does Not Increase DNA Damage

**Figure 7:**
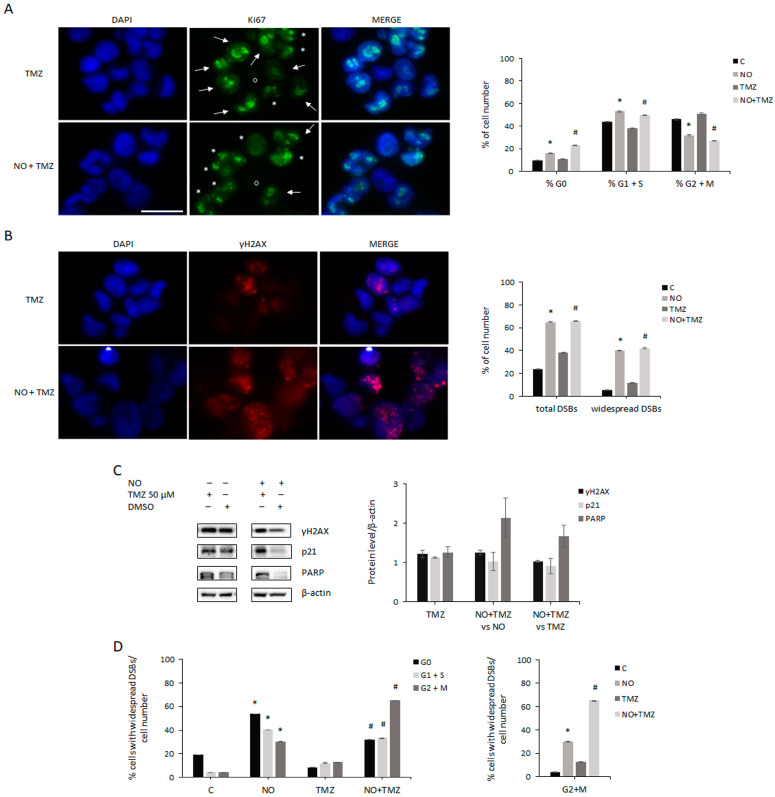
Effects of cotreatment on cell cycle progression and DNA damage. (**A**) Representative immunofluorescence of GSCs after 7 days of TMZ (top) and TMZ plus NO cotreatment (bottom) showing nuclei, Ki67 foci, and merged images. Ki67 staining patterns indicate different stages of the cell cycle: asterisks indicate cells in G1/S phases, arrows indicate cells in G2 phase, and circles indicate cells in G0 (Ki67^−^ cells). The scale bar = 50 µm is the same in every image. The same analysis was also performed on GSCs untreated or treated with NO for 14 days (shown in Figure 3A). The graph shows the changes induced by the indicated treatments on the percentage of cells in different phases of the cell cycle and in G0, evaluated through the peculiar pattern of Ki67. The values were obtained from the analysis of at least 15 fields and 100–150 cells for each sample. All the values represent the mean ± SEM. * *p* < 0.05 vs. untreated cells; # *p* < 0.05 vs. TMZ-treated cells. (**B**) Representative immunofluorescence staining of GSCs after 7 days of TMZ (top) and TMZ plus NO cotreatment (bottom) showing nuclei, γH2AX foci, and merged images. The scale bar = 50 µm is the same as panel A. The same analysis was performed on GSCs untreated and after 14 days of NO treatment (shown in Figure 4B). The graph shows changes induced by the different treatments on the percentage of cells containing total DSBs (left bars) and cells containing only widely spread DSBs staining throughout the nuclei (right bars). The values were obtained from the analysis of at least 15 fields and 100–150 cells for each sample. All the values are represented as mean ± SEM. * *p* < 0.05 vs. untreated cells; # *p* < 0.05 vs. TMZ-treated cells. (**C**) Representative Western blots showing the expression of markers of the DNA damage response and repair after 21 days of exposure to 50 µM TMZ, or DMSO used at the same dilution, in the presence (right) or absence (left) of NO (TMZ and DMSO administration started 7 days after NO). The expression of each protein was normalized to β-actin. The graph shows the level of each protein after TMZ administration alone (left bars) and the changes induced by NO plus TMZ cotreatment with respect to NO (middle bars) and TMZ (right bars). The mean ± SEM of the densitometric analysis of 2 experiments is shown. (**D**) The left graph shows the percentage of cells with widespread DSBs in each phase of the cell cycle and in G0, identified through the peculiar pattern of Ki67, normalized to the percentage of cells in the same cell cycle phase. The values were obtained from the analysis of at least 15 fields and 100–150 cells for each sample. The values are represented as mean ± SEM. * *p* < 0.05 vs. untreated cells; # *p* < 0.05 vs. TMZ-treated cells. The right graph shows only the percentage of cells in G2 + M phase with widespread DSBs after the indicated treatments (highlighted from the left graph).

We evaluated whether cell proliferation inhibition induced by NO plus TMZ cotreatment could be due to cell cycle progression deregulation. Therefore, GSCs were pre-treated with NO for 7 days and further treated with NO plus TMZ for an additional 7 days, when 50% inhibition on cell proliferation was observed with respect to NO exposure alone, and compared to the effects individually induced by TMZ after 7 days as well as by NO after 14 days of administration. The specific pattern of Ki67 expression in the different phases of the cell cycle was analyzed through immunofluorescence (Figure 7A). As summarized in the graph, consistent with the lack of changes in GSC proliferation, TMZ treatment did not induce relevant effects in cell cycle progression with respect to untreated cells. Differently, when the effects induced by NO plus TMZ cotreatment were compared to TMZ alone, we observed that the fraction of G0 cells was more than doubled, the percentage of cells in the G1/S phase increased by 30%, and G2/M cells decreased by 50%. As shown in the graph of Figure 7A, when comparing these results with the effects induced by individual NO administration (which were also shown in Figure 3A), it was evident that combined treatment induced more potent effects than NO alone, mainly affecting the percentage of cells in G0 and G2/M phases, which further increased and decreased, respectively. We then evaluated whether NO plus TMZ cotreatment could modify the rate of NO-induced DNA damage. As shown in Figure 7B, the analysis of total γH2AX foci, irrespective of foci intensity and size, revealed that TMZ administration increased the percentage of γH2AX-positive cells compared with untreated cells. However, NO plus TMZ treatment did not increase the percentage of GSCs with DSBs generated by NO alone. Similarly, combined treatment did not increase the percentage of cells presenting widely diffuse γH2AX staining caused by NO exposure, although TMZ alone enhanced the number of heavily damaged nuclei (Figure 7B, right). Next, we performed Western blot analysis to evaluate the level of γH2AX, p21, and PARP after 21 days of TMZ exposure in the presence or absence of NO. Consistent with the above findings, TMZ alone had weak effects on the basal level of γH2AX, p21, and PARP (Figure 7C, left blots and left triple bars in the graph), whereas NO plus TMZ co-treatment markedly increased PARP levels compared to both NO and TMZ singly administered (Figure 7C). Finally, we delved into the lack of DNA damage increase upon NO plus TMZ administration, evaluating the distribution of nuclei containing widely spread γH2AX foci in each cell cycle phase, as determined by Ki67 staining. As shown in Figure 7D (left graph), in untreated cells the highest percentage of fully damaged cells were in G0, whereas TMZ treatment weakly induced DNA damage irrespective of the cell cycle phase. Differently, NO administration strongly increased DNA damage mainly in the G1/S and G2/M phases, and to a lesser extent in the G0 phase, vs. untreated cells (9,4-, 7,4-, and 2,8-fold, respectively). Interestingly, NO plus TMZ cotreatment primarily increased the percentage of severely damaged nuclei in G2/M cells, although G0 and G1/S cells also presented a high percentage of nuclei with widespread DSBs when compared to TMZ-treated cells (5,1-, 3,9-, and 2,8-fold, respectively). Altogether, these results indicate that NO plus TMZ administration induced a 40% increase in severely damaged G0 cells compared to NO administration alone, whereas no further damage was observed in the other phases of the cell cycle. Interestingly, when only fully damaged cells in the G2/M phase were selected from the previous analysis (Figure 7D, right graph), a close similarity with respect to the trend of the percentage of cells in G0 upon the different treatments (shown in Figure 7A) could be observed.

### 2.8. NO Plus TMZ Combined Treatment Inhibits Constitutive Caspase 3 Activation

**Figure 8:**
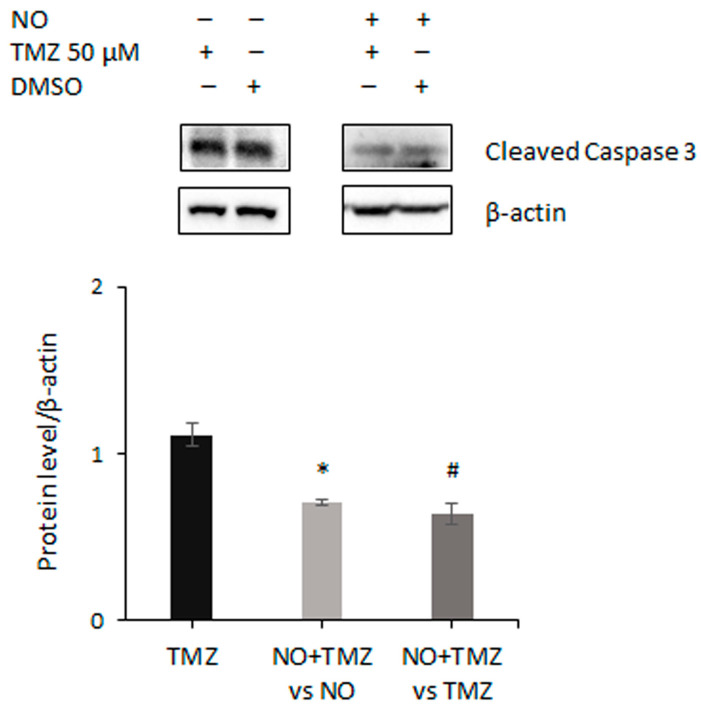
NO plus TMZ cotreatment decreases active caspase 3. Representative Western blot showing the expression of active caspase 3 in GSCs after 21 days of treatment with 50 µM TMZ, or DMSO at the same dilution, in the presence (right) or absence (left) of NO. TMZ and DMSO administration was started 7 days after NO treatment. The level of active caspase 3 was normalized to β-actin and the mean ± SEM of the densitometric analysis of 2 experiments is recorded. Left bar shows the effect of TMZ alone calculated with respect to DMSO. The middle bar shows changes induced by TMZ plus NO cotreatment with respect to NO single administration. The right bar shows the effect of combined treatment with respect to TMZ administration alone. * *p* < 0.05 vs. NO-treated cells; # *p* < 0.05 vs. TMZ-treated cells.

In order to investigate the mechanisms underlying the decrease in GSC proliferation, a possible apoptosis induction following NO plus TMZ treatment was also evaluated. The analysis of Annexin V expression on the cell membrane in GSCs treated with 50 µM TMZ for 21 days in the presence of NO did not support this hypothesis, in agreement with the results of the immunofluorescence analyses shown in Figure 7 which did not reveal nuclear fragmentation, neither after administration of TMZ alone nor after NO plus TMZ cotreatment. To explore this further, we also evaluated the level of activated caspase 3. As shown in Figure 8, while TMZ treatment did not induce changes in the protein level, combined treatment unexpectedly reduced constitutively activated caspase 3, compared to NO and TMZ administration alone.

## 3. Discussion

A malignant tumor is a complex structure possessing wide cellular heterogeneity. It also includes tumor-initiating cells and their descendants at different stages of differentiation [62]. Tumor heterogeneity has important therapeutic implications and may cause the failure of some therapeutic regimens. This negative behavior is critical in GBM, a lethal disease without effective therapy [1]. Indeed, the current treatment with TMZ has only transient benefits, and ultimately glioblastomas acquire resistance and relapse [63,64], as the treatment does not impact GSCs [7,8,9,59]. However, it has been reported that combining chemotherapy and other specific molecules could be more effective [12,61,65]. NO is a gaseous molecule playing multiple roles in cancer. However, its role in GSCs’ biology is still scarcely investigated. With the aim to enhance the knowledge about GSCs’ response to novel putative therapeutic agents, some of the signaling pathways underlying the effects of NO and TMZ on the stem cell compartment of GBM were elucidated in this study. As widely reported, NO-induced effects depend on its dose, as nanomolar and micromolar concentrations usually exert pro-tumorigenic or anti-proliferative actions, respectively [33,34]. In agreement with these findings, we observed that a low NO concentration (50 µM) supported GSC proliferation, whereas too-high concentrations (200 and 400 µM) induced cytotoxic effects. Indeed, over the IC50 concentration, we mainly observed quick cell death consequent to a dramatic increase in DNA damage, despite cells’ attempts to activate repair mechanisms. Unlike too-high toxic concentrations, 100 µM NO treatment induced widespread effects by affecting several pathways. Importantly, prolonged 100 µM NO administration prevented the expansion of the GSCs’ subpopulation, acting on both GSCs’ self-renewal and proliferation. This action of NO was paralleled by an increased rate of cell death, even if it was too poor to justify cell proliferation arrest. Interestingly, in the presence of NO, most of the cells expressed the stemness marker SOX2, although a small population of SOX2^−^ cells arose after NO administration. The evidence of these non-stem cells was consistent with the presence of single cells in NO-treated cell cultures that were no longer able to self-renew and generate neurospheres but were “committed” to die. Notably, after 200 µM NO exposure, we could observe a larger SOX2^−^ cell population, consistent with the reduced number of spheres and increased cell mortality. So far, our data suggest that NO may also reprogram GSCs by inducing a shift from symmetric to asymmetric cell division. Previous reports have demonstrated the plasticity of stem cells in general and also of GSCs which may reduce the number of daughter stem cells in favor of an increased number of differentiated cells, depending on environmental signals [66,67]. This hypothesis seems in agreement with our findings, as, following NO exposure, a population of non-stem cells developed and died as they could not survive in stem cell culture conditions. The plasticity of GSCs was further confirmed by the observation that, after NO withdrawal, GSCs were able to recover the symmetric proliferative capacity typical of untreated cells.

According to previous reports, we could observe that NO caused persistent DNA damage able to interfere with correct cell cycle execution [42,43,53], thus reducing GSC proliferation rate. Indeed, NO treatment induced an accumulation of cells in S-phase, reduced the number of cells able to exit the cell cycle, and increased the percentage of non-proliferating cells. However, a parallel increase in PARP level suggests a possible strategy of GSCs to resist massive DNA damage. Therefore, after 100 µM NO exposure, GSCs experienced tolerable DNA damage, resulting in a state of equilibrium where cells neither regressed nor expanded. Interestingly, our results suggest that NO could prevent overall GBM growth, as NO administration also inhibited the expansion of differentiated glioblastoma cells. However, it was evident that NO activated different signaling pathways in the differentiated and stem-like cells, also inducing cell-type-specific effects. Indeed, U87MG cells appeared more sensitive to NO treatment than GSCs, since the recovery of cell proliferation was deeply compromised after treatment withdrawal. NO-induced DNA damage had more severe and persistent effects on U87MG than on GSCs. Indeed, after prolonged treatment, U87MG cells showed DNA damage and cell-type-specific activation of the p53/p21 pathway (consistent with cell cycle arrest) but no activation of repair mechanisms. Consistently, after NO withdrawal, cells still showed partially altered pathways associated with DNA damage, which impeded the recovery of proliferation capacity. Indeed, repair mechanisms slowly appeared only after NO deprivation, indicating that U87MG cells cannot counteract NO action as long as the treatment is administered. On the contrary, GSCs confirmed their strong resistance and resilience as NO withdrawal quickly restored DNA integrity prior to treatment, allowing the usual proliferation capacity to recover.

It is known that cells with widespread DNA damage usually undergo apoptosis, thus preventing the passage of damaged DNA to the daughter cells [68,69]. However, contrary to several reports showing that NO administration may provoke apoptosis [45,70,71], NO-treated GSCs did not show apoptosis induction, which could be at least in part associated with the p53 negative phenotype of the cells. Indeed, the tumor suppressor p53 is a recognized activator of apoptosis, senescence, and growth arrest, aiming to prevent tumor formation related to conditions inducing cellular stress [72]. The unexpected observation that GSCs constitutively expressed activated caspase 3, recognized as a critical executor of the apoptotic cascade [55], suggests that these cells resist signals that usually induce programmed cell death. It appears that NO-exposed GSCs survive to DNA damage and do not undergo apoptosis, but part of the population become quiescent or does not complete cell cycle division, resulting in GSCs’ expansion prevention. These exciting findings suggest that GSCs have developed efficient mechanisms to tolerate insults that usually would eliminate cells.

As an alternative tumor suppressor program, severe DNA damage may induce cells not undergoing apoptosis to enter into an irreversible proliferation arrest termed cellular senescence [73]. Consistent with the demonstration that NO may induce senescence in different cell types [74], our results showed that long-term NO administration might induce senescent features in GSCs, such as proliferation prevention, DNA damage foci induction, and generation of enlarged cells. Senescent cells’ recognition by the immune system, including when senescence is induced by drug treatment, represents a key strategy in cancer eradication [75,76]. Consistent with the observation that glioma cells grown in 3D spheroid culture are more resistant to NK cytotoxic activity than cells in traditional 2D flat culture [77], GSCs were reported to be poorly susceptible to lysis mediated by NK cells [78]. These findings, together with our observations suggesting possible NO-induced senescence in GSCs, propose a novel and exciting pathway through which NO combined with immunotherapy, could eliminate GSCs. This hypothesis deserves a specific focus, and is consistent with a recent strategy that oncologists are pursuing, which aims to combine chemotherapy and immunotherapy to make the tumor more visible to the immune system.

We wondered whether the stalemate situation in which GSCs are found, after long-term NO treatment, could make them susceptible to combined treatment. Indeed, it has been shown that NO may sensitize tumor cells to radio- and chemotherapy [45,46] and that combined NO/TMZ treatment inhibited tumor growth in vivo [45]. These findings prompted us to investigate whether NO-survived GSCs with severe DNA damage, which impedes cell proliferation, could become sensitive to TMZ. Importantly, we observed that, whereas TMZ alone was ineffective, TMZ plus NO administration, following NO-conditioning, further reduced GSC proliferation. Consistent with a previous report showing that cell cycle arrest was not a significant event of TMZ-induced antitumor activity in glioma stem cells [59], we observed that TMZ administration could affect cell cycle progression only in the presence of NO, increasing NO-induced inhibitory effects. In particular, reduced cell proliferation with combined NO/TMZ cotreatment could be ascribed, at least in part, to the increased percentage of cells entering the G0 phase with respect to NO exposure alone. Our findings suggest that, although a part of damaged cells may complete the cell cycle, the entity of DNA damage may prevent their entry into a new cell cycle. Therefore, it appears evident that, although NO and TMZ may act through different paths, they both prevent the expansion of the GSCs’ subpopulation mainly through a cytostatic action. However, the observed increase in cell death may also contribute to reducing the GSCs’ subpopulation. Interestingly, our results also highlighted that, although both NO and TMZ alone could increase the percentage of severely damaged cells, their co-administration did not result in additive or synergistic effects. These findings suggest that GSCs can limit excessive DNA damage occurrence. It is known that TMZ induces DNA damage through specific residue methylation, but the action of the O6-methylguanine DNA methyltransferase (MGMT) repairs the DNA lesion [45]. Accordingly, the GSCs used in our experiments express MGMT [79], which may be accountable for the resistance to TMZ administration, and consistent with the absence of p53 [80]. Significantly, the absence of MGMT in p53-positive U87MG cells [81] accounts for their sensitivity to TMZ exposure. It appears, therefore, that MGMT and PARP may contribute to the repair of TMZ- and NO-induced DNA damage, respectively, thus explaining why two DNA-damaging agents do not sum their effects when administered in combination. The additional observation that PARP level increased only after NO/TMZ cotreatment further highlighted GSCs’ self-preservation skills.

In this scenario, our findings that p53-negative GSCs were insensitive to apoptosis when exposed to both TMZ alone and NO/TMZ in combination are unsurprising. Indeed, this is consistent with the notion that p53 wildtype glioma cells are more sensitive than p53 defective cells to TMZ-induced apoptosis [82], and with previous reports showing that TMZ administration was scarcely effective on apoptosis induction in GSCs [59]. In this regard, our results show critical novel findings concerning the unexpected action of caspase 3 in GSCs upon combined treatment, as we found a significant reduction in constitutively activated protein fragment. However, cleaved caspase 3 was also associated with functions different from apoptosis [83]. In agreement with a previous report, which showed that caspase 3 seems to be involved in cell proliferation [84], our results showed that NO plus TMZ cotreatment reduced both cell proliferation and expression of the activated form of the protein, suggesting that GSCs could be an unexpected cell model in which caspase 3 plays a role in cell proliferation. These findings unveil a novel level of complexity in GSCs’ biology that deserves further investigation.

As far as we know, this is the first study investigating the effects of exogenous NO in GSCs. Eyler and co-workers [85] showed that NO generated through highly expressed iNOS in GSCs promoted cell growth and tumorigenicity, suggesting iNOS silencing as a promising approach to inhibit tumor growth. However, subsequent studies have shown that, at high concentrations, NO administration inhibits iNOS expression in GBM cells [45], suggesting a negative feedback loop in iNOS regulation. These findings may explain the opposite effect observed in our experimental approach due to the elevated NO concentration used.

## 4. Materials and Methods

### 4.1. Cell Cultures and Treatments

Patient-derived BT168 GSCs [50,86] were cultured in serum-free medium consisting of DMEM/F12 (Sigma-Aldrich, Saint Louis, MO, USA) supplemented with 20 ng/mL EGF, 20 ng/mL bFGF, 2% B27 w/o vitamin A (Thermo Fisher Scientific, Waltham, MA, USA), 1% glutamine and 1% antibiotics (Sigma-Aldrich) in an incubator under standard culture conditions. GSC proliferation generated floating rounded spheres with well-defined borders, which were kept until they were suitably sized to ensure cell health, and well spaced from each other to avoid sticking. Twice a week, subconfluent spheres were centrifuged at 400 rpm for 20 min and then incubated with accutase (Thermo Fisher Scientific) for 15 min at 37 °C. Single GSCs were centrifuged at 800 rpm for 5 min and seeded in fresh medium. U87MG cells (from the American Type Culture Collection, Manassas, VA, USA) were routinely grown in DMEM supplemented with 10% FBS, 1% glutamine, and 1% antibiotics (Sigma-Aldrich). DETA/NO, TMZ and DMSO were from Sigma-Aldrich. DETA/NO spontaneously releases NO in aqueous media with a half-life of 20 h. Therefore, it was dissolved in sterile water and supplied to the cells every 48 h. TMZ was dissolved in DMSO and supplied every 72–96 h. Parallel to TMZ treatment, control cells were always supplied with DMSO, diluted as TMZ.

### 4.2. Cell Proliferation and Death

In all the experiments, 150,000 GSCs/mL of culture medium were seeded. The next day, cells were treated with NO or vehicle. The treatment was repeated for the duration of the experiment as described above. To evaluate the effect of TMZ, 150,000 GSCs/mL of culture medium were plated and, the next day, treated with TMZ or DMSO. The treatments were repeated for the duration of the experiment as described above. In the experiments with NO plus TMZ cotreatment, 150,000 GSCs/mL of culture medium were seeded, and were treated the next day with vehicle or NO. Seven days later, TMZ or DMSO were added to both vehicle and NO-treated cells. NO, TMZ and DMSO treatments were repeated for all the duration of the experiment. To note, GSCs received NO treatment alternately as single cells and during neurospheres’ growth, whereas TMZ administration always coincided with neurosphere dissociation and seeding of single GSCs. At each passage, when spheres were dissociated to single cell suspension, GSCs were plated again at the starting density, in both untreated and treated samples (with different dilutions). According to the experimental design, after 3, 7, 14, and 21 days, spheres were dissociated and single cells were stained with 0.4% Trypan blue to evaluate the number of both alive and dead cells through the use of a Burker counting chamber. To reveal the presence of necrotic cells in intact spheres, untreated and NO-treated neurospheres were stained with Trypan blue (1:4 in stem cell medium) for 4 h, then placed in PBS and photographed using a phase contrast microscope. IC50 value was calculated using GraphPad Prism v5.01 (GraphPad Software, San Diego, CA, USA). U87MG cells were seeded at about 8000 cells/cm^2^. The next day, cells were treated with NO, water, TMZ, or DMSO, according to the experimental design. The treatments were repeated, as described above, for the duration of each experiment. Twice a week, U87MG cells were enzymatically detached, and control cells were always seeded at the starting density in fresh medium. After 7 days of treatment, treated cells were seeded at a double density than control cells, due to the progressive effects of treatment on cell proliferation and size. At the appropriate time points, subconfluent cells were tripsinized, stained with 0.4% Trypan blue and counted using a Burker counting chamber.

### 4.3. Cell Morphology

The morphology of both floating neurospheres and adherent U87MG cells was observed under an Axiovert 40 inverted microscope (Zeiss, Oberkochen, Germany). Cell pictures were taken with a ZOE cell imager (Bio-Rad, Hercules, CA, USA).

### 4.4. Immunofluorescence Analysis

GSCs were seeded at 150,000 cells/mL of culture medium. On the cells treated with NO or vehicle for 3, 7, and 14 days (as described above) immunostaining for SOX2, Ki67, and γH2AX was performed. Moreover, on GSCs treated for 7 days with TMZ alone and in combination with NO (as described above), immunostaining for Ki67 and γH2AX was also performed. Briefly, at each time point, neurospheres were dissociated, and GSCs were fixed in 4% paraformaldehyde and cytocentrifuged onto a clean slide using a Shandon cytocentrifuge at 800 rpm for 5 min, permeabilized in 0.3% Triton in PBS for 5 min, and then incubated in PBS containing 10% BSA for 30 min. Samples were immunoassayed by using the mouse anti-SOX2 (Santa Cruz Biotechnology, Dallas, TX, USA), rabbit anti-γH2AX, and mouse anti-Ki67 (Cell Signaling Technology, Danvers, MA, USA) antibodies, all diluted 1:100 in PBS, O/N at RT. Primary antibodies were detected by 1 h incubation with FITC-conjugated anti-mouse (1:100, Jackson ImmunoResearch, Ely, UK) or Cy3-conjugated anti-rabbit (1:300, Thermo Fisher Scientific). The slides were mounted in Vectashield with DAPI (Vector) to stain the DNA and reduce fluorescence fading. All images were captured using a CoolSnap HQ CCD camera (Photometrics, Tucson, AZ, USA) connected to a Zeiss Axioplan fluorescence microscope equipped with an HBO 100 W mercury lamp. At least 15 fields and 100–150 cells for each sample were analyzed by three independent individuals.

### 4.5. Western Blot Analysis

In all the experiments, 150,000 GSCs/mL of culture medium were seeded. Protein expression was analyzed in GSCs, following treatment with NO and TMZ, singly and in combination, as described above, as well as in U87MG cells after NO treatment. Intact neurospheres were pelleted at 400 rpm for 20 min and solubilized in Laemmli buffer on ice (5000 cells/µL of buffer). Adherent U87MG cells were scraped and solubilized in Laemmli buffer on ice (5000 cells/µL of buffer). Before lysis, the number of cells in each sample was obtained by counting a parallel series of replicates. Cell lysates were boiled for 10 min and the supernatants were collected after centrifugation at 13,000 rpm for 30 min at 4 °C. Equal volumes of protein extracts were subjected to 12% SDS-PAGE and electrophoretically transferred to nitrocellulose membranes which were probed with primary antibodies. Anti-SOX2 and -p53 antibodies were from Santa Cruz Biotechnology, anti-PCNA was from Abcam (Cambridge, UK), anti-γH2AX and -p21 were from Cell Signaling Technology, anti-caspase 3 was from Millipore (Temecula, CA, USA), anti-PARP was from DB Pharmingen (Franklin Lakes, NJ, USA), and anti-β-actin was from Sigma-Aldrich. After incubation with the appropriate secondary antibody conjugated to peroxidase (Sigma-Aldrich), SuperSignal West Pico plus or SuperSignal West Femto chemiluminescent systems (Thermo Fisher Scientific) were used for band detection. Images were captured at a ChemiDoc XRS+ Gel Imaging System (Bio-Rad). Band densitometry was performed by ImageJ 1.47v software.

### 4.6. Flow Cytometry Analysis

GSCs were seeded at 150,000 cells/mL of culture medium. The next day, cells were treated with NO or vehicle, and grown as described above. At the adequate time points, control and NO-treated spheres were dissociated, then the cells were counted and resuspended in PBS and fixed for 2 h at 4 °C in cold 70% ethanol. Thereafter, GSCs were incubated for 30 min at room temperature with 50 µg/mL propidium iodide (PI) in PBS containing 0.5 mg/mL RNAse (Sigma-Aldrich) and immediately analyzed using a FACSCanto (BD Biosciences, San Jose, CA, USA). Analysis of the cell cycle distributions was performed using the Dean–Jett–Fox model in FlowJo V10 Cytometric Analysis Software (BD Biosciences).

### 4.7. Apoptosis Analysis

Apoptosis occurrence was investigated by the Annexin V-FITC apoptosis detection kit (Abcam), according to the manufacturer’s instructions. Briefly, 150,000 GSCs were seeded and treated with NO alone and in combination with TMZ, as described above. After 21 days of NO treatment and NO/TMZ cotreatment, neurospheres were dissociated and 100,000 cells were resuspended in 500 µL of Binding Buffer. Five µL of Annexin V-FITC antibody and 5 µL of PI were added. Cells were incubated 5 min in the dark, then placed onto a glass slide, covered with a glass coverslip and observed under an Axio-observer3 inverted microscope (Zeiss, Oberkochen, Germany).

### 4.8. Statistical Analysis

Data were analyzed using GraphPad Prism v5.01 software and are presented as mean values ± SEM from at least three independent experiments. Statistical significance between treated and control cells was determined using the paired Student’s *t*-test, while the unpaired *t*-test was used when two different treatment groups were compared. *p* values of <0.05 were considered significant.

## 5. Conclusions

Given the multifaceted challenge of GBM, the need for optimizing existing therapies and identifying additional treatments to enhance current therapeutic strategies is clear. Therefore, the primary focus of our study revolved around deciphering the pathways that confer GSCs’ resistance to treatments. To this aim, we evaluated the effects of prolonged NO exposure, which elicited significant cytostatic effects leading to the curtailment of GSCs’ expansion. Notably, GSCs conditioned with NO displayed sensitivity to TMZ, exhibiting further suppression of cell proliferation, expansion of the quiescent cell subpopulation, and increased cell death. Our findings suggest that, while administering NO alone may not eliminate the GSCs’ subpopulation, it could be a valuable therapeutic tool when used with TMZ. Indeed, this combination might effectively overcome TMZ resistance in the stem-like cell subpopulation, thus dramatically limiting GSC proliferation. Furthermore, given that differentiated glioblastoma cells also displayed sensitivity to both NO and TMZ, the combined treatment could be effective against the whole tumor. However, further in-depth molecular studies are necessary to explore the mechanisms underpinning the combined NO/TMZ treatment to better understand its antiproliferative effects. Moreover, our hypothesis could be further investigated by developing tools for site-specific NO release or hydrogel-mediated NO delivery [87,88]. In addition, in vivo studies are crucial to determine if NO/TMZ treatment might possess a tangible antineoplastic effect capable of delaying GBM relapses. Nonetheless, our research serves as a foundational stepping stone for formulating innovative therapeutic strategies geared towards extending the survival of GBM patients by controlling the tumor stem-like cell subpopulation. This possibility aligns with a novel perspective that aspires to a scenario where cancer, although incurable, can be managed over extended periods, effectively transforming it into a chronic condition.

## Data Availability

Not applicable.

## Supplementary Materials

The following supporting information can be downloaded at: https://www.mdpi.com/article/10.3390/ijms241411286/s1.
