# Supplementary material for: Nitric Oxide Prevents Glioblastoma Stem Cells’ Expansion and Induces Temozolomide Sensitization
Source: Int J Mol Sci. 2023 Jul 10;24(14):11286. doi: 10.3390/ijms241411286 (PMC10379318; doi:10.3390/ijms241411286)
Supplement: Supplementary file 1 [file ijms-24-11286-s001.zip › ijms-2470137-supplementary.pdf]

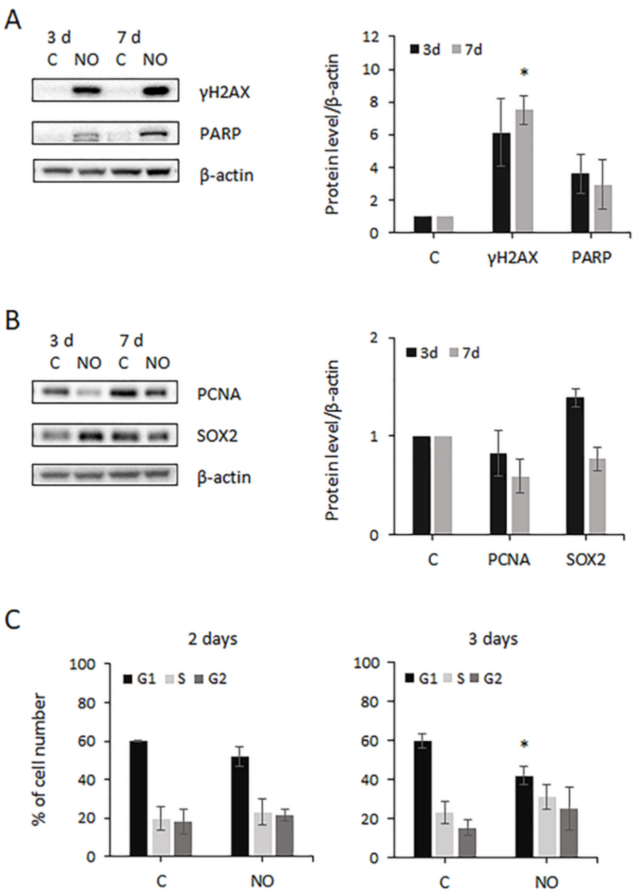

Figure S1. Toxic effects of 200 μM NO administration. Representative Western blots showing the expression of (A) γH2AX and PARP as wells as (B) PCNA and SOX2 in untreated cells (C) and after 200 μM NO treatment at the indicated times. The expression level of each protein was normalized to β-actin. The graphs show the fold changes with respect to the protein level in untreated cells at each time point, to which a value equal to 1 was arbitrarily assigned. The mean ± SEM of at least 3 experiments is shown. *, $p < 0.05$ vs. control cells. (C) Flow cytometry analysis of the cell cycle progression in untreated cells and after 2 and 3 days of NO exposure. Results represent mean ± SEM from 3 experiments. *, $p < 0.05$ vs. control cells.

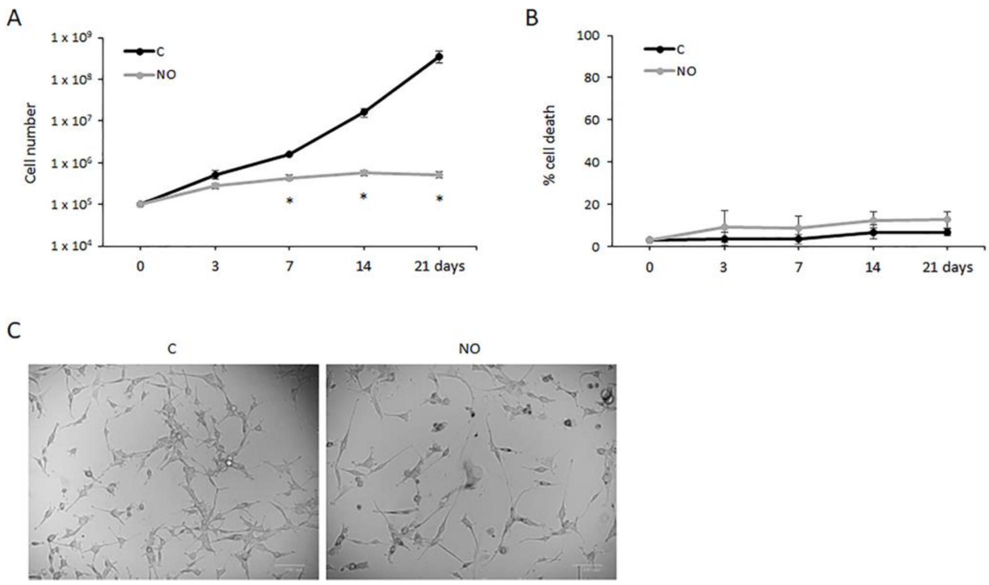

Figure S2. NO prevents the proliferation of U87MG glioblastoma cells. Effects on cell proliferation (A) and death (B) induced by treatment with 100 μM NO at the indicated times. Results represent the mean ± SEM from 3 experiments. *, $p < 0.05$ vs. control cells. (C) Photographs of untreated (left) and 21 days NO-treated cells (right) were taken under a phase contrast microscope (10x).

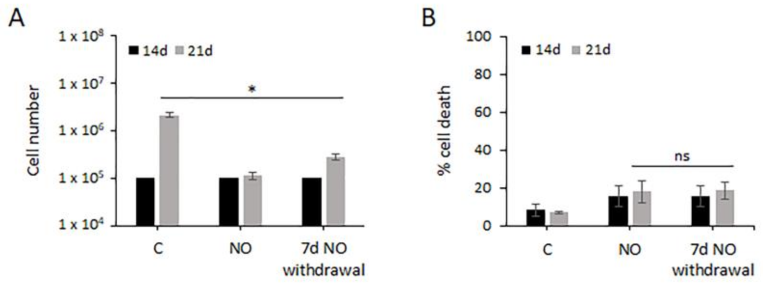

Figure S3. NO impairs U87MG cell proliferation recovery. The effects on U87MG cell proliferation (A) and death (B) induced by 7 days of NO withdrawal after 14 days of treatment are shown (right bars) and compared to cells exposed to NO for 21 days (middle bars) and untreated cells (left bars). Results represent the mean ± SEM from 3 experiments. *, $p <$ 0.05 vs. untreated cells.

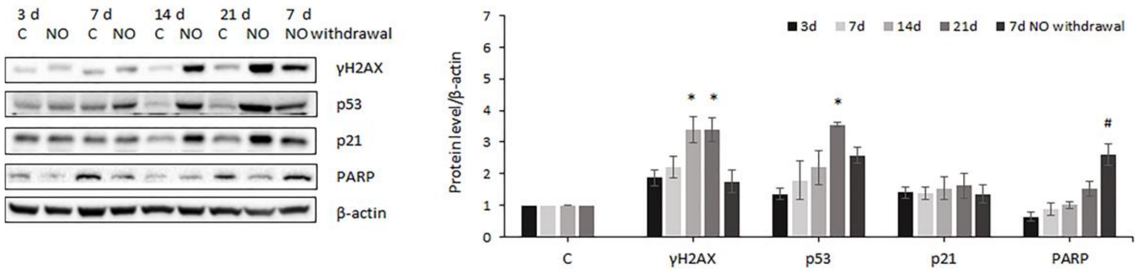

Figure S4. NO induces DNA damage in U87MG glioblastoma cells. Representative Western blots showing the level of DNA damage and repair markers analyzed in untreated cells (C) as well as after 3, 7, 14, and 21 days of NO treatment (NO) and after 14 days of NO exposure followed by 7 days of treatment withdrawal (right lane) in U87MG cells. The densitometric analysis of each protein was normalized to that of β-actin and shown in the graph with respect to the protein level in untreated cells at each time point, to which a value equal to 1 was arbitrarily assigned. The mean ± SEM of the densitometric analysis of at least 3 experiments is shown. *, $p < 0.05$ vs. untreated cells; #, $p < 0.05$ vs. 21 days NO-treated cells.

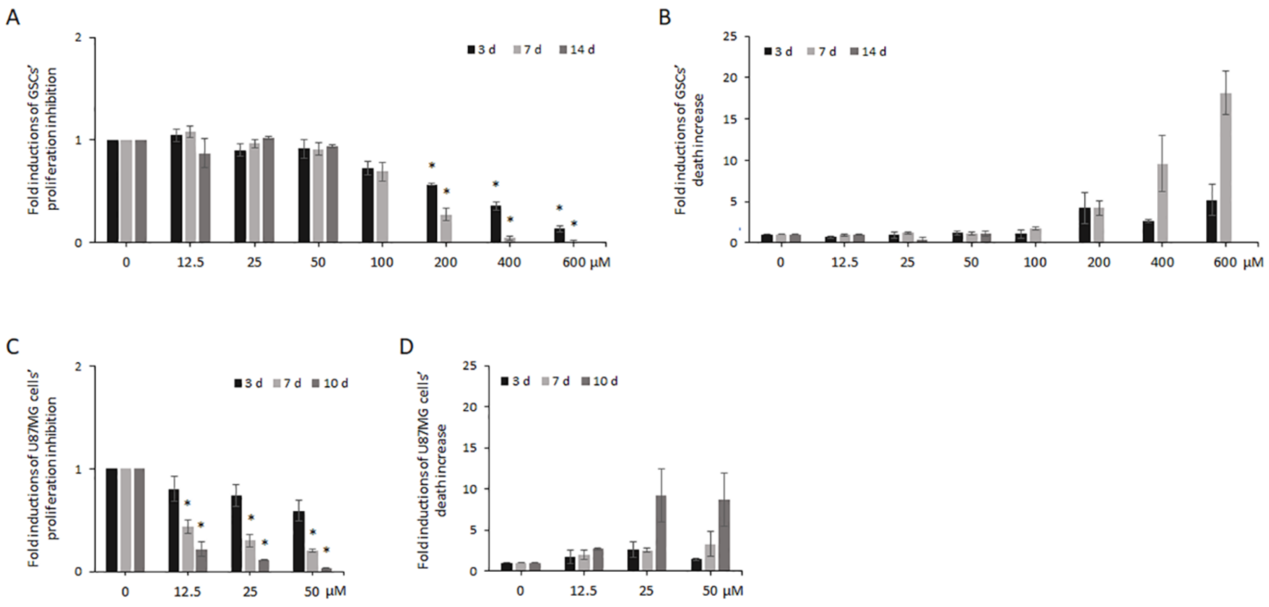

Figure S5. U87MG cells are more sensitive than GSCs to Temozolomide (TMZ). Effect of several concentrations of TMZ on proliferation (A and C panels) and death (B and D panels) in GSCs (upper panels) and U87MG cells (lower panels), evaluated after treatments at the indicated times. At each concentration, TMZ-induced effects were normalized to those induced by DMSO at the same dilution. All the values represent the mean ± SEM of at least 3 experiments. **p* < 0.05 vs. untreated cells.
